# Anti-inflammatory and antitumor activities of the chloroform extract and anti-inflammatory effect of the three diterpenes isolated from *Salvia ballotiflora* Benth

**DOI:** 10.1186/s12906-020-03179-w

**Published:** 2021-01-07

**Authors:** Nimsi Campos-Xolalpa, Ángel Josabad Alonso-Castro, Elizabeth Ortíz-Sanchez, Juan Ramon Zapata-Morales, Marco Martin González-Chávez, Salud Pérez

**Affiliations:** 1 Doctorado en Ciencias Biólogicas y de la Salud, Universidad Autónoma Metropolitana-Xochimilco, Calzada del Hueso 1100, Col. Villa Quietud, Delegación Coyoacán, CP 04960 Ciudad de México, Mexico; 2 Departament of Farmacia, División de Ciencias Naturales y Exactas, Universidad de Guanajuato, Noria Alta S/N C.P, 36050 Guanajuato, GTO Mexico; 3 Instituto Nacional de Cancerología, Subdirección de Investigación Básica, Av. San Fernando 22, Belisario Domínguez, 14080 CDMX, CO Mexico; 4 Centro de Investigación y Estudios de Posgrado, Facultad de Ciencias Químicas, Universidad Autónoma de San Luis Potosí, Av. Dr. Manuel Nava 6, Zona Universitaria, 78210 San Luis Potosí, SLP, Mexico; 5 Department of Sistemas Biológicos, Universidad Autónoma Metropolitana-Xochimilco, Calzada del Hueso 1100, Col. Villa Quietud, Delegación Coyoacán, CP 04960 Ciudad de México, Mexico

**Keywords:** *Salvia ballotiflora*, Anti-inflammatory activity, Antiarthritic activity, Antitumoral activity, Chloroform extract

## Abstract

**Background:**

Drugs used for the treatment of diseases associated with chronic inflammation, such as cancer and rheumatoid arthritis have the potential to cause undesirable side-effects, which might result in patients ending treatment prematurely. However, plants are a viable option for the treatment of inflammatory diseases. In this study, we assessed the in vivo and *in vitro* anti-inflammatory activity, and the antitumor effects of the chloroform extract of *Salvia ballotiflora* (ECL). The pro-apoptotic effects of ECL in CT26 cells were also determined.

**Methods:**

The chloroform extract of *Salvia ballotiflora* (ECL) was standardized using 19-deoxyicetexone (DEOX) as a phytochemical marker. The anti-inflammatory activity of ECL was determined on acute and chronic inflammatory models using the TPA-induced mouse ear edema assay. The antitumor activity of ECL was evaluated by the subcutaneous inoculation of CT26 cells on the back of Balb/c mice. *In vitro* CT26 cell death induced by ECL was determined by Annexin V/propidium iodide staining assay using flow cytometry. ECL and the diterpenes isolated from the chloroform extract included 19-deoxyicetexone (DEOX), icetexone (ICT), and 7,20-dihydroanastomosine (DAM), which were tested in LPS-stimulated J774A.1 macrophages to quantify pro-inflammatory cytokine levels. The *in vitro* anti-arthritic activity of ECL was determined using the bovine serum protein (BSP) denaturation assay.

**Results:**

ECL exerted anti-inflammatory activities in acute (84% of inhibition, 2 mg/ear) and chronic models (62.71%, at 100 mg/kg). ECL showed antitumor activity at 200 mg/kg and 300 mg/kg, reducing tumor volume by 30 and 40%, respectively. ECL (9.5 μg/mL) induced *in vitro* apoptosis in CT26 cells by 29.1% (48 h of treatment) and 93.9% (72 h of treatment). ECL (10 μg/ml) decreased levels of NO (53.7%), pro-inflammatory cytokines IL-6 (44.9%), IL-1β (71.9%), and TNF-α (40.1%), but increased the production of the anti-inflammatory cytokine IL-10 (44%). The diterpenes DEOX, ICT, and DAM decreased levels of NO (38.34, 47.63, 67.15%), IL-6 (57.84, 60.45, 44.26%), and TNF-α (38.90, 31.30, 32.83%), respectively. ECL showed *in vitro* antiarthritic activity (IC_50_ = 482.65 μg/mL).

**Conclusions:**

ECL exhibited anti-inflammatory and anti-tumor activities. Furthermore, the diterpenes DEOX, DAM, and ICT showed anti-inflammatory activity by reducing levels of NO, TNF-α, and IL-6.

## Background

Inflammation is a complex biological response of the immune system to repair damage caused by injury including chemical, biological, physical, or mechanical agents [1]. Depending on its duration, inflammation can be acute or chronic. Acute inflammation is a beneficial response, whereas chronic inflammation can induce tissue damage [2]. Chronic inflammation can also lead to the progression of many diseases, such as rheumatoid arthritis and cancer [2].

Cancer is a process of uncontrolled cell growth that forms tumors and produces damage to surrounding tissues due to the inflammatory response. In most cases, premalignant lesions, inflammatory diseases, and cancer are treated with different types of drugs, however, most of these compounds cause severe side-effects [3]. Active compounds obtained from medicinal plants used in traditional medicine are considered alternative treatments for several diseases, including cancer and inflammatory-related diseases [4]. The S*alvia* genus, with more than 900 species, is composed of many plants with different biological activities including insecticidal, antifungal, cytotoxic, and anti-inflammatory, among others [5].

*Salvia ballotiflora* Benth (Lamiaceae), commonly known in Mexico as “mejorana”, is an endemic plant from Northern Mexico and the Southern United States of America used in folk medicine for baths during pregnancy [6]. From this plant species, two diterpene quinones have been isolated, icetexone (ICT) and conacytone [7] as well as other diterpenes, such as 19-deoxyicetexone (DEOX), 19-deoxyisocetexone (DIC), and 7,20-dihydroanastomosine (DAM), among others [8]. Other isolated compounds from *Salvia ballotiflora* include 7α-acetoxy-6,7-dihydroicetexone, and anastomosine, these two compounds have shown cytotoxic activity against the cancer cell lines U251 and SKLU-1, and an anti-inflammatory effect on acute ear edema induced by TPA in mice [9]. Additional, some studies have found that different diterpenes exhibited an important anti-inflammatory effect [10]. Recently, DEOX, DIC, and DAM were obtained from the hexane-washed chloroform extract of *S. ballotiflora* (ECL), and the cytotoxic activity of ECL was determined against the cancer cell lines A549, CT26, HeLa, and MCF7 [6]. These results show that *S. ballotiflora* and some of its metabolites have cytotoxic activity, and anti-inflammatory activity. However, there is not reports about the the anti-inflammatory effect of ECL, DEOX, DAM and ICT, mechanism of action, and the ECL effect on cancer tumor. For these reasons, we evaluated the anti-inflammatory effect in acute and chronic models and antitumor activities of ECL, including the anti-inflammatory effects *in vitro* of ECL and three of the isolated diterpenes (DEOX, ICT, and DAM). Lastly, the pro-apoptotic effects of ECL in CT26 cells were also determined.

## Methods

### Materials

Fetal bovine serum (FBS), Dulbecco’s Modified Eagle Medium (DMEM), antibiotics, 3-(4,5-dimethylthiazol-2-yl)-2,5-diphenyl tetrazolium bromide (MTT), indomethacin (IND), diclofenac sodium (DICLO), polyvinylpyrrolidone (PVP), Griess reagent (GR), and 12-O-tetradecanoylphorbol-13-acetate (TPA) were purchased from Sigma Aldrich (St. Louis, MO, USA). The murine colorectal cancer cell line CT26 and the murine J774A.1 macrophages were purchased from ATCC (Manassas, VA, USA), whereas cisplatin (CDDP) was acquired from PISA Laboratory (PISA™ Pharmaceutics, Guadalajara, Mexico).

### Plant material

*Salvia ballotiflora* Benth was collected in Las Comadres, Municipality of Guadalcazar within the state of San Luis Potosi, Mexico in August 2017. A voucher specimen was preserved in the Isidro Palacios Herbarium of the Autonomous University of San Luis Potosí (SLPM43014). A taxonomist (José García Pérez) identified the plant material. *S. ballotiflora* is not an endangered species, and for this reason, a collection permit is not required by the Secretariat of Ecology and Environmental Management of San Luis Potosi (SEGAM-SLP).

### Extract preparation

The dried and ground aerial parts (500 g) of *S. ballotiflora* were extracted with 3 L of chloroform at the boiling point for 4 h. The mixture was filtered and the solvent was evaporated to dryness under reduced pressure. The solid material was then washed with warm hexane (ECL) and a 5.07% yield was obtained.

### Isolation of DEOX, ICT, and DAM

The diterpenes were isolated from ECL by open column chromatography using a mixture of hexane-ethyl acetate as described earlier [6].

### Analysis by GC-MS

A mixture of 5 mg ECL or 1 mg DEOX, 1 mL of isooctane and 100 μL of bis(trimethylsilyl) trifluoroacetamide with 10% of trimethylsilyl chloride was heated at 100 °C for 30 min.

The ECL analysis was performed with a gas chromatography/mass spectrometer (Agilent Technology, model 6890 N) connected to a mass detector model 5973 with a DB-5HT capillary column. The injector temperature was set at 200 °C. The initial oven temperature (250 °C) was held for 2 min, then the temperature was increased at a rate of 15 °C/min up to 325 °C and maintained for 3 min. The splitless injection was performed at a ratio of 1:100 and the injector temperature was 325 °C. The spectrum was determined at 70 eV. DEOX was identified in ECL and extrapolation of this diterpene was performed on the standard curve (500–31 ppm). We use DEOX for the extract standardization because this diterpene is the most abundant of the three diterpenes in ECL [6].

### Animals

One hundred fifty-two Male CD1 and forty-eight Balb/c mice (20–25 g, 6–7 weeks of age) from the animal facility of the Autonomous Metropolitan-Xochimilco University, were used. The experimental protocol (140) was approved by the Research Bioethics Committee of the Autonomous Metropolitan-Xochimilco University. All experiments were performed in compliance with the Mexican Official Norm for Animal Care and Handling (NOM-062-ZOO-1999). Mice were maintained with free access to food (Lab Diet 5001) and water ad libitum. Animals were housed at 24 °C ± 1 °C with 12 h light/dark cycles. The mice were acclimatized in the laboratory for 2 weeks prior to the experiments, which began at 8:00 am. After experiments, conscious animals were sacrificed in a CO_2_ chamber.

### Acute toxicity test

The Lorke methodology [11] was followed to evaluate the acute toxicity of ECL. Briefly, ECL in PVP (1:4) from 400 to 5000 mg/kg, was orally administered to mice as a single dose (*n* = 10 per test group) and compared with the negative control group. Mice were monitored, under open-field conditions every 24 h, during 72 h. At the end of 72 h, the number of animal deaths was recorded and the rest were sacrificed conscious in a CO_2_ chamber. Then, biopsies were obtained to identify possible damage to the stomach, spleen, liver, and kidneys.

### Acute anti-inflammatory activity: TPA-induced mouse ear edema

A TPA (2.5 μg) solution in acetone (20 μL) was topically administered to both, the inner and outer surfaces of the right ear of mice, and acetone (vehicle) was administered to both surfaces of the left ear of mice from the ECL, positive (IND), and negative study groups (*n* = 8 per group). After 30 min, 2.0 mg/ear ECL or IND dissolved in acetone, was topically administered to the right ear, whereas the vehicle was applied to the left ear. After 6 h, conscious animals were sacrificed in a CO_2_ chamber, and 6 mm portions of the central sections of both ears were obtained [12]. The weight of these tissue portions were recorded and the percent inhibition of ear edema was determined as follows:

$$ \%\mathrm{Inhibition}=\left(\frac{\left({W}_t-{W}_{nt}\right)\mathrm{control}-\left({W}_t-{W}_{nt}\right)\mathrm{treated}}{\left({W}_t-{W}_{nt}\right)\mathrm{control}}\right)100 $$

*W*_*t*_: weight of treated ear, W_nt_: weight of non-treated ear.

### Chronic anti-inflammatory activity

ECL (12.5, 25, 50, or 100 mg/Kg), 8 mg/kg IND (positive control) or vehicle were orally administered to each group of 8 mice. Samples were dissolved in saline solution with PVP (1:4). After 30 min, a TPA (2.5 μg) solution in acetone (20 μL) was topically applied to the inner and outer surfaces of the right ear of mice, whereas acetone alone was applied to both surfaces of the left ear. The topical application of TPA was carried out in the 1st, 3rd, 5th, 7th, and 9th day of the experiment. On the last day, conscious animals were sacrificed in a CO_2_ chamber, and the weight of 6 mm plugs from the central portion of both ears was recorded. The percent inhibition of edema was determined as described above [13].

### Cell culture conditions

J774A.1 murine macrophages and CT26 murine carcinoma cells were maintained in DMEM supplemented with 10% fetal bovine serum (FBS) and antibiotics (100 U/mL penicillin and 100 pg/mL streptomycin). Cell cultures were grown at 37 °C and 5% CO_2_.

### Cell viability assay

The viability of the macrophages treated with ECL, DEOX, ICT, or DAM was determined. In a 96-well plate, 5000 cells/well were plated and the extract and the three diterpenes were applied at concentrations of 1 to 100 μg/mL for 24 h. Then, 10 μL of MTT (5 mg/mL) solution was placed in each well; 4 h later, the medium was removed and the formazan crystals formed in each well were dissolved with 100 μL of DMSO [14]. The absorbance was read at 540 nm in a BioRad spectrophotometer. The IC_50_ was calculated by linear regression.

### Determination of Nitric Oxide (NO) and cytokine levels

In 6 well-plates, 5 × 10^5^ macrophages J774A.1 were seeded per well. Macrophages were stimulated with 5 μg/mL LPS. After 2 h, cells were treated with ECL (10 μg/mL), DAM and ICT (25 μg/mL), DEOX (20 μg/mL), or reference drug (IND 17.1 μg/mL), and (culture medium with vehicle only). After 24 h, supernatants were collected for the quantification of NO and levels of IL-1β, IL-6, IL-10, and TNF-α, using commercial enzyme-linked immunosorbent assays (ELISA), following the manufacturer’s instructions (PROMEGA). The absorbance was recorded at 405 nm. For the quantitation of NO production, 100 μL of supernatant was mixed with 100 μL of Griess reagent in a 96-well plate. The reaction mixture was incubated at 37 °C for 30 min and the absorbance was measured at 540 nm with an ELISA reader (Bio-Rad). A 100% nitric oxide production was considered for the LPS group [15].

### Membrane stabilization property

Blood samples were collected, maintaining aseptic conditions, from healthy human volunteers that did not consume non-steroidal anti-inflammatory drugs (NSAIDs), steroids, or oral contraceptives for 2 weeks prior to the experiment. Blood samples were washed with an equal volume of Alsever’s solution (2% dextrose, 0.8% sodium citrate, 0.05% citric acid, and 0.42% sodium chloride in water), and centrifuged at 3000 rpm for 10 min; then packed cells were washed three times with Alsever’s solution.

A 5% erythrocyte suspension was mixed with different concentrations (25–400 μg/mL) of ECL or DICLO prepared in PBS buffer. Distilled water and PBS buffer were used as negative controls. All samples were incubated at 37 °C for 30 min and centrifuged at 3500 rpm for 5 min [16]. The optical density was read at 450 nm. The percent protection was calculated with the following equation:

$$ \% Protection=100-\left(\frac{optical\ density\ of\ Test\ sample}{Optical\ density\ of\ Control}\ X\ 100\right) $$

### *In vitro* anti-arthritic activity

The bovine serum protein (BSP) denaturation method [17] was used to evaluate *in vitro* anti-arthritic activity. A BSP solution (5% w/v aqueous solution) was prepared as follows: 0.45 mL of BSP solution and 0.05 mL of the drug treatment. The ECL concentrations were 25, 50, 100, 200, 500, 750, and 1000 μg/mL and the DICLO treatment included the same concentrations as ECL, and distilled water was used as a control. Samples were heated at 37 °C for 20 min and the temperature was increased to 57 °C for 3 min. After cooling, 2.5 mL PBS was added to the solutions. The absorbance was measured at 560 nm. The control represents 100% protein denaturation. The percentage of protein denaturation inhibition was estimated as follows:

$$ \%\mathrm{of}\ \mathrm{protein}\ \mathrm{denaturation}\ \mathrm{inhibition}=100-\left(\frac{\mathrm{Optical}\ \mathrm{density}\ \mathrm{of}\ \mathrm{Test}-\mathrm{Optical}\ \mathrm{density}\ \mathrm{of}\ \mathrm{Control}}{\mathrm{Optical}\ \mathrm{density}\ \mathrm{of}\ \mathrm{Test}}\ \mathrm{x}\ 100\right) $$

### Apoptosis assay

CT26 cells were seeded in 100 mm^2^ culture plates (2 × 10^6^ cells/plate). After 24 h, cells were incubated with CDDP (1.4 μg/mL), ECL (9.5 μg/mL), or DMSO at a final concentration of 0.01% (vehicle group). After treatment (48 h or 72 h), cells were detached with EDTA solution in 1X PBS, washed and centrifuged at 4500 rpm for 5 min. Cells were stained with the Annexin V/propidium iodide kit (Dead Cell Apoptosis Kit with Annexin V FITC and PI, for flow cytometry. Invitrogen) and read in a FacScan flow cytometer (BD Bioscience, USA). Data from 20,000 acquired events were analyzed using the Weasel cytometry analysis software v.2.6.1.

### Antitumor assay

Balb/c mice were injected subcutaneously in their backs with CT26 cells (6.5 × 10^5^) [18]. Eight days later, groups of 8 tumor-bearing mice (volume of 50 to 100 mm^3^) received oral doses of ECL (100, 200, and 300 mg/kg), dissolved in saline solution with PVP (1:4), 1 mg/kg CDDP (p.o. and i.p.) for 22 days, or 0.1 mL of saline solution. Tumors were measured using a Vernier, and size (in mm^3^) was calculated as follows:

$$ Tumor\ volume=\frac{length\ast width\ast height}{2} $$

At the end of the experiments, animals were sacrificed in a CO_2_ chamber, and tumors were excised and weighed.

### Statistical analysis

Data are expressed as the mean ± S.E.M. When indicated, Student’s t-test and ANOVA followed by Dunnett’s test, were used. A *P* < 0.05 was considered significant. Results were processed using the SPSS software from IBM.

## Results

### Analysis by GC-MS

ECL was standardized using DEOX as a phytochemical marker. The calibration curve was linear from 31 to 500 ppm (R^2^ = 0.9933). The results showed that ECL contains 0.2 mg/g (0.02%) of DEOX.

### Acute toxicity test

Animals administered with ECL at doses of 625, 1250, 2500, and 5000 mg/kg presented a lethal dose (LD_50_) higher than 5000 mg/kg; however, ECL at doses of 625 mg/kg showed visible damage in the kidneys, spleen, stomach, and liver of mice. ECL at 400 mg/kg showed no visible damage to organs. Therefore, doses lower than 400 mg/kg ECL were used for subsequent studies.

### Anti-inflammatory activity of ECL in a mouse model of TPA-induced ear edema

The anti-inflammatory activity of ECL was evaluated on acute TPA-induced ear edema in mice at doses of 2 mg/ear of indomethacin (IND) or ECL. The extract significantly inhibited inflammation by 84.79 ± 4.07%, with similar activity to IND (89.16 ± 6.4%). No significant differences (*n* = 8, *p* > 0.05) were shown between ECL and IND.

### Anti-inflammatory activity of ECL in mouse ear edema induced by multiple applications of TPA

The chronic anti-inflammatory activity of ECL was evaluated in a model of multiple applications of TPA. ECL showed dose-dependent anti-inflammatory activity (ED_50_ = 45.33 mg/kg). ECL at doses of 12.5 mg/kg (26.43%) and 25 mg/kg (30.07%), showed low anti-inflammatory action, whereas ECL at doses of 50 mg/kg (53.28%) and 100 mg/kg (62.71%) presented a comparable anti-inflammatory effect to IND (8 mg/kg) (64.32%). These results show that ECL exerts an anti-inflammatory activity in a 9-day assay (Table 1).

**Table 1:** Anti-inflammatory activity of ECL in ear edema induced by multiple applications of TPA in mice

GROUP	Dose (mg/kg)	% Inhibition of inflammation
**VEHICLE**	0	0 #
**IND**	8	64.32 ± 2.47 *
**ECL**	12.5	26.43 ± 7.12 * #
	25	30.07 ± 2.41 * #
	50	53.28 ± 2.21 *
	100	62.71 ± 2.30 *

Results expressed as mean ± S.E.M. (*n* = 8). Statical analysis were performed using one way Anova test followed by Dunnet’s comparison test. Significante difference *p* ≤ 0.05 versus (*) vehicle and (#) IND group

### Cell viability in macrophages

**Fig. 1:**
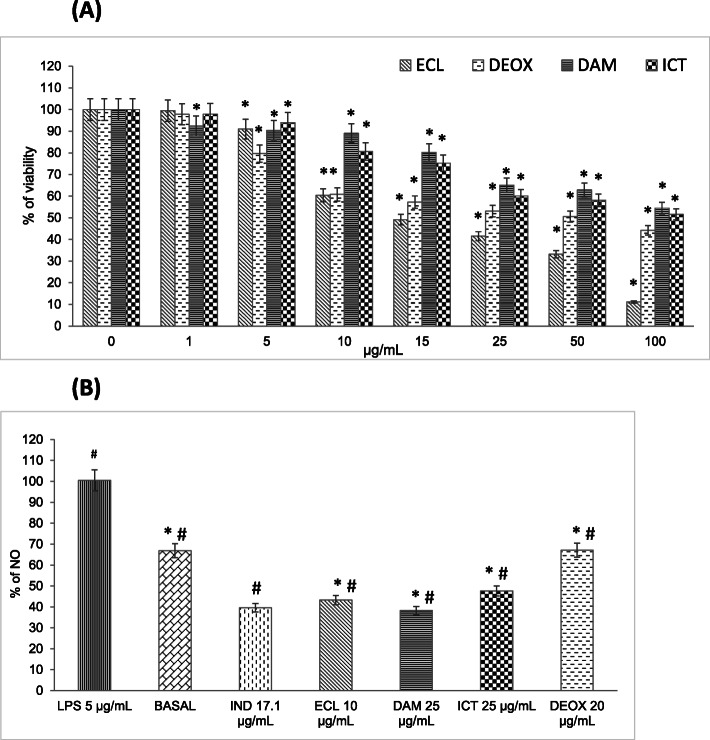
**a** Viability of macrophages treated with ECL, DAM, ICT and DEOX at 1–100 μg/mL (*n* = 10). **b** Effects of ECL (10 μg/mL), DAM and ICT (25 μg/mL), DEOX (20 μg/mL), and 17.1 μg/mL IND in LPS-stimulated macrophages on % production of NO (*n* = 4). Data were expressed as mean ± S.E.M. Values were significantly different compared with the (*) vehicle and (#) IND group using the Dunnett’s test: *p < 0.05

The IC_50_ values were as follows: 16.97 ± 2.04 μg/mL (ECL); 116.17 ± 7.33 μg/mL (DAM); 114.97 ± 7.09 μg/mL (ICT), and 54.57 ± 2.85 μg/mL (DEOX). Then, we used concentrations of the extract and compounds that did not affect 60% cell viability of J774A.1 macrophages (Fig. 1a). Therefore, we used 10 μg/mL ECL, 25 μg/mL DAM and ICT, and 20 μg/mL DEOX in subsequent experiments.

### Determination of NO levels

Figure 1b shows the percentage of NO produced by macrophages stimulated with LPS and treated with ECL, DAM, ICT, or DEOX. The findings showed that ECL, DAM, ICT, and DEOX inhibited NO production by 56.74, 61.66, 52.37, and 32.85%, respectively. The reduction in NO levels by ECL and DAM was similar to the reference drug, 17.1 μg/mL IND (60.41%). However, DAM and ICT showed a low effect on NO inhibition.

### Levels of inflammatory cytokines

**Fig. 2:**
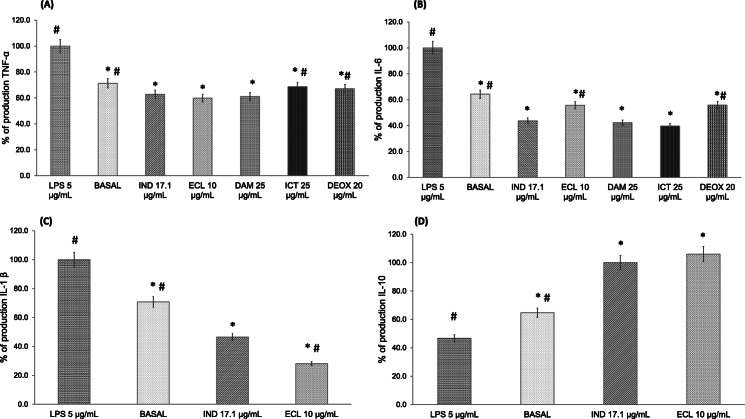
Effects of ECL (10 μg/mL), DAM and ICT (25 μg/mL), DEOX (20 μg/mL), and 17.1 μg/mL IND in LPS-stimulated macrophages on % of production of (**a**) TNF-α and (**b**) IL-6. Effects of ECL (10 μg/mL) on % of production of (**c**) IL-1β and (**d**) IL-10. The values are the mean ± s.e.m. of three independent experiments (*n* = 4). Values were significantly different compared with the (*) vehicle and (#) IND group using the Dunnett’s test: *p < 0.05

The production of pro-inflammatory (IL-6, TNF-α, and IL-1β) and anti-inflammatory (IL-10) cytokines was assessed in LPS-stimulated macrophages treated with ECL (Fig. 2). In addition, IL-6 and TNF-α levels were determined in the presence of DAM, ICT, and DEOX (Fig. 2a, b).

ECL diminished the production of the pro-inflammatory cytokines by 71.9% for IL-1β. ECL, DAM, ICT, and DEOX inhibited the production of IL-6 by 44.9, 57.84, 60.45, and 44.26%, respectively, and of TNF-α by 40.1, 38.90, 31.30, and 32.83%, respectively. In addition, ECL increased the production of the anti-inflammatory cytokine IL-10 by that more 80%, with similar activity induced by IND (17.1 μg/mL).

### Membrane stabilization properties

The *in vitro* anti-inflammatory activity of ECL was determined using the human red blood cell (HRBC) membrane stabilization method [16]. ECL evaluated at 25, 50, 100, 200, and 400 μg/mL showed membrane protection by 85.13 ± 0.16%, 87.52 ± 0.16%, 89.37 ± 0.37%, 90.97 ± 0.33%, and 91.078 ± 0.13% respectively. The IC_50_ value was 0.144 ± 0.0038 μg/mL. The results obtained with ECL were comparable to those obtained with DICLO at concentrations of 25, 50, 100, 200, and 400 μg/ml, which showed membrane stabilization in 89.71 ± 0.29%, 91.64 ± 0.31%, 92.10 ± 0.63%, 94.82 ± 0.17%, and 96.60 ± 0.21%, respectively. The IC_50_ value was 0.138 ± 0.0003 μg/mL.

### *In vitro* anti-arthritic activity

The percentages of inhibition of bovine serum protein denaturation induced by ECL and DICLO are shown in Table 2. ECL presented antiarthritic activity in a concentration-dependent manner. DICLO, at the same concentrations, diminished protein denaturation (Table 2). The *in vitro* anti-arthritic activity effect of ECL (IC_50_ = 482.65 ± 12.67 μg/mL) was lower in comparison to DICLO (IC_50_ = 123.39 ± 14.65 μg/mL).

**Table 2:** *In vitro* anti-antiarthritic activity of ECL using the bovine serum protein denaturation method

Group	Concentration (μg/mL)	% denaturation	% Inhibition of denaturation
**Control**	–	100	0
**DICLO**	1000	9.85 ± 2.80	90.15 ± 2.81
	750	25.63 ± 7.29	74.37 ± 7.29
	500	33.27 ± 0.01	66.73 ± 0.01
	200	42.04 ± 4.09	57.96 ± 4.09
	100	51.90 ± 2.79	48.10 ± 2.79
	50	61.58 ± 0.26	38.42 ± 0.26
	25	65.89 ± 0.01	34.11 ± 0.01
**ECL**	1000	27.00 ± 6.61	72.99 ± 6.61
	750	42.20 ± 4.07	57.79 ± 2.75
	500	49.32 ± 2.75	50.67 ± 2.75
	200	54.60 ± 4.35	45.39 ± 4.35
	100	61.38 ± 3.11	38.61 ± 3.11
	50	65.82 ± 3.03	34.17 ± 3.03
	25	70.16 ± 0.10	29.84 ± 0.10

The values are the mean ± S.E.M of three independent experiments

### ECL induces apoptosis in CT26 cells

**Fig. 3:**
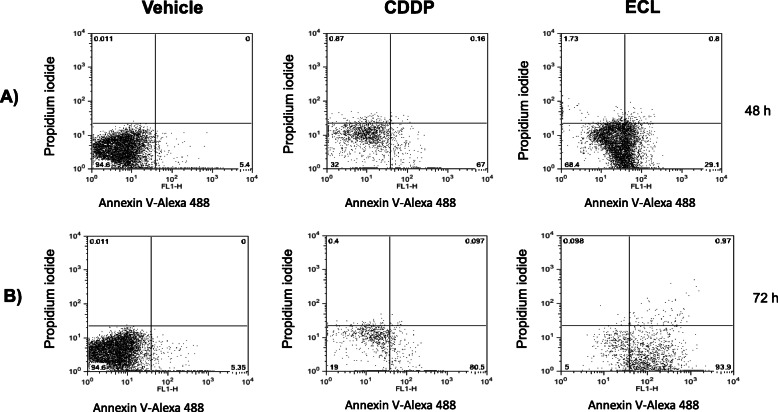
CT26 cells were incubated for 48 h (**a**) and 72 h (**b**) treated with vehicle (DMSO 0.01%), CDDP (1.4 μg/mL) and ECL (9.5 μg/mL). The apoptosis assay with Annexin V and Propidium Iodide was used, recording 20,000 events. The percentage of cells in each quadrant is found in the corner. The cells in the right quadrants (Annexin V+/PI – and Annexin V+/PI+) are considered as apoptotic cells. Data are representative of three independent experiments

Apoptosis as a biological effect of ECL was evaluated in murine colon carcinoma CT26 cells. Flow cytometry assays showed that 29.1% of CT26 cells were in apoptosis after 24 h of ECL treatment (Fig. 3a). Furthermore, higher levels of apoptosis in CT26 cells were found after 72 h of treatment (93.3%), compared to the CDDP treatment (Fig. 3b), used as a positive treatment control (80.5%).

### Antitumor assay

**Fig. 4:**
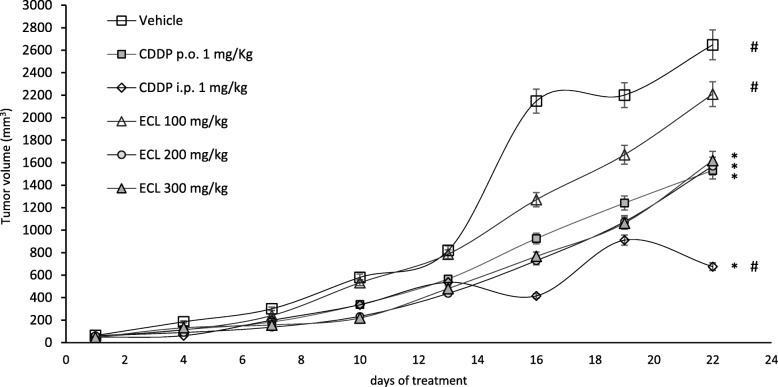
Tumor volume (mm^3^) during 22 days of treatment. Data were expressed as mean ± s.e.m. (*n* = 8). Data were expressed as mean ± S.E.M. Values were significantly different compared with the (*) vehicle and (#) CDDP p.o. group using the Dunnett’s test: *p < 0.05

The antitumor activity of ECL was evaluated in Balb/c mice bearing CT-26 tumor for 22 days. The tumor volume from the vehicle group showed the largest tumor size among the experimental groups (2647.73 ± 464.48 mm^3^). ECL at doses of 200 mg/kg (1569.88 ± 386.70 mm^3^) and 300 mg/kg (1618.87 ± 295.70 mm^3^) decreased tumor volume in a similar pattern as with 1 mg/kg CDDP p.o. (1531.20 ± 236.80 mm^3^) (Fig. 4).

At the end of the treatment, tumors from the vehicle group were the heaviest (6.01 g). ECL decreased tumor weight or in a dose-dependent manner: 100 mg/kg (34.86%), 200 mg/kg (38.14%), and 300 mg/kg (51.10%). The reference drug, 1 mg/kg CDDP p.o. decreased tumor weight by 57.09% (Table 3). ECL showed a ED_50_ of 291.51 mg/kg.

**Table 3:** Weight of tumors in mice after 22 days of administration of the different study groups

Study groups	Tumor weight (g)	% of weight decrease
**Vehicle**	6.01 ± 0.99 * ^#^	0 ^#^
**CDDP (1 mg/Kg) i.p.**	1.25 ± 0.59 * ^#^	79.21 * ^#^
**CDDP (1 mg/Kg) p.o.**	2.58 ± 0.42 *	57.09 *
**ECL 100 mg/Kg p.o.**	3.92 ± 0.68 * ^#^	34.86 * ^#^
**ECL 200 mg/Kg p.o.**	3.72 ± 0.65 *	38.14 * ^#^
**ECL 300 mg/Kg p.o.**	2.94 ± 0.55 *	51.10 *

Data were expressed as mean ± S.E.M. (*n* = 8). Values were significantly different compared with the (*) vehicle and (#) CDDP p.o.group using the Dunnett’s test: *p < 0.05

## Discussion

We found that the yield of DAM, ICT, and DEOX was lower than 0.005% [6], and as a result, only few experiments with these compounds were performed [9]. Therefore, the in vivo models, quantitation of IL-1β and IL-10 in LPS-stimulated macrophages, anti-arthritic activity, membrane stabilization assay, the apoptosis and the antitumor assay were assessed only with ECL standardized with DEOX as the phytochemical marker.

This study shows for the fist time, the anti-inflammatory activity of the extract and the three diterpenes, and the antitumor effect of the extract.

ECL presented in vivo anti-inflammatory activities in both acute and chronic assays. ECL diminished mouse acute TPA-induced ear edema by 84.79%, with a similar activity compared to IND treatment (89.16%). The inflammation produced by topical applications of TPA is mediated by protein kinase C, the formation of reactive oxygen species, prostaglandins, and the production of phospholipase A_2_ [19]. Therefore, a strategy used to control inflammation is the inhibition of some of these inflammatory mediators. According to the results, ECL reduced acute and chronic inflammation induced by TPA. This indicates that ECL might inhibit the production of inflammatory mediators. The chronic TPA-induced mouse ear edema is caused by the production of infiltrating leukocytes, which eventually causes tissue damage [20]. ECL at 100 mg/kg showed a reduction (62.71%) of chronic TPA-induced mouse ear edema with activity comparable to the IND positive control group (64.32%). These findings clearly indicate that ECL exerts in vivo anti-inflammatory activity.

It is also demonstrated that ECL, DAM, ICT, and DEOX showed *in vitro* anti-inflammatory actions in LPS-stimulated macrophages. It is well known that LPS promotes the production of NO and cytokines in macrophages [21]. Nitric oxide is a substance present in different biological processes, such as inflammation. NO plays an important role in the pathogenesis of inflammation and its production can be used as a measure of the progression of inflammation [22]. The release of NO to the extracellular space induces the activation of chemokines, cytokines, and endothelial-leukocyte adhesion molecules, with the subsequent vasodilation and the formation of reactive nitrogen species that produce tissue damage [23]. Therefore, NO inhibitors are useful tools in the treatment of inflammatory diseases [24]. The concentrations of ECL and its diterpenes that showed cell viability higher than 60%, diminished NO levels in LPS macrophages.

During the inflammatory process, there is an increase in vascular permeability and cell migration, such as macrophages and neutrophils, which are related to the synthesis of prostaglandins and cytokines, and the increase of NO release. Cezarotto and collaborators [25] found that ICT diminished neutrophil migrations, which is associated with its anti-inflammatory activity. We found that ICT inhibited NO production, which suggests a decrease in cell migration.

During the inflammatory process, the production of cytokines, such as IL-1β, IL-6, and TNF-α by macrophages occurs to repair damaged tissues [1]. TNF-α, a cytokine involved in acute inflammatory processes, regulates the levels of other pro-inflammatory cytokines like IL-6, which is involved in the induction and perpetuation of early inflammation [26]. IL-1β is another pro-inflammatory cytokine that plays an important role in host-defense responses against injury induced by infections and pain progression [27]. IL-1β increases tissue damage during chronic diseases [28]. IL-10 is an anti-inflammatory cytokine that limits the host immune response by preventing damage to the host and contributing to the maintenance of tissue homeostasis [1]. Thus, the inhibition of pro-inflammatory cytokines (IL-1, IL-6, and TNF-α), and the increase in the production of the anti-inflammatory cytokine IL-10 by ECL, may be an effective strategy in the treatment of inflammatory conditions. DAM, ICT, and DEOX also diminished IL-6 and TNF-α levels, which suggests that the anti-inflammatory activity is due to these diterpenes. ECL significantly increased IL-10 production with similar activity in comparison with IND.

The hypotonic solution exerts hemolytic effects related to fluid accumulation in cells and the subsequent breaking of cell membranes. Compounds with membrane-stabilizing effects might inhibit phospholipase production by diminishing the levels of some inflammatory mediators [29]. The erythrocyte membrane is analogous to the liposomal membrane and the prevention of hypotonicity-induced HRBC membrane lysis is a direct measurement of the anti-inflammatory effect of drugs [30]. ECL showed a high effect (IC_50_ = 0.144 ± 0.0038 μg/mL) on membrane-stabilization of erythrocytes. This effect was comparable to DICLO (IC_50_ = 0.138 ± 0.0003 μg/mL). Therefore, this extract may act as a potent anti-inflammatory agent.

Rheumatoid arthritis is one of the most common inflammatory diseases with an increasing incidence worldwide. During the development of this disease, protein denaturation occurs, resulting in the formation of auto-antigens and the consequent development of joint inflammation [31]. In this study, ECL decreased heat-induced protein denaturation, suggesting that ELC could exert anti-arthritic activity.

Persistent inflammation can cause DNA damage, leading to the initiation and progression of cancer [32]. Chronic inflammation is caused by the constant activation of the immune system, which inevitably results in the accumulation of genetic and epigenetic aberrations, and the subsequent malignant transformation, also induced by the participation pro-inflammatory cytokines, transcription factors, tumor suppressor genes, and oncogenes [33]. These findings might suggest that ECL could be used as an anti-inflammatory and antitumor agent, since this extract decreases in vivo chronic inflammation.

Chemotherapy relies on the inhibition of proliferation and the induction of apoptosis in cancer cells [34]. Apoptosis is the process of programmed cell death, considered an important component of various processes, including normal cell turnover, proper development and functioning of the immune system, embryonic development, chemical-induced cell death, cell growth, and cytoskeletal rearrangement [35]. Alterations in apoptosis cause neurodegenerative diseases, ischemic damage, autoimmune disorders, and many types of cancer [36]. At 72 h of treatment, ECL induced apoptosis in CT26 cancer cells with similar activity as with CDDP, the reference drug. Previously, it was found that ECL exerted cytotoxic activity on CT26 cells and other cancer cell lines [6]. Tumor suppression is a hallmark of cancer progression [33]. In this work, ECL showed the ability to induce *in vitro* apoptosis in CT26 cells and reduce the growth of tumors in Balb/c mice bearing CT26 tumors. Since ECL decreased tumor growth in mice, this effect was similar to that obtained when the animals were orally administered with CDDP. Then, it is possible that ECL might inhibit proliferation of CT26 cells. Therefore, ECL may be a potential cytotoxic and antitumor agent in the treatment of colorectal cancer. The anti-inflammatory activity of natural products is related to their cytotoxic and antitumor actions [37]. In this context, ECL as well as DAM, ICT, and DEOX could be considered important candidates for the treatment of inflammatory diseases and cancer.

## Conclusion

For the first time, ECL has shown *in vitro* and in vivo anti-inflammatory effects and *in vitro* anti-arthritic activities. ECL induced apoptosis in CT26 cells and showed antitumor activity. In LPS-stimulated macrophages, ECL, DAM, ICT, and DEOX inhibited the production of NO, and decreased pro-inflammatory cytokine levels. Furthermore, ECL increased the levels of the anti-inflammatory cytokine IL-10. ECL is a protective agent against both membrane lysis and protein denaturation.

## Data Availability

The data associated with this study is available from corresponding author.

## Abbreviations

ECL: Chloroform extract of *S. ballotiflora*
ICT: Icetexone
DEOX: 19-deoxyicetexone
DIC: 19-deoxyisocetexone
DAM: 7,20-dihydroanastomosine
FBS: Fetal bovine serum
DMEM: Dulbecco Modified Eagle Medium
MTT: Antibiotics, 3-(4,5-dimethylthiazol-2-yl)-2,5-diphenyl tetrazolium bromide
IND: Indomethacin
DICLO: Diclofenac sodium
PVP: Polyvinylpyrrolidone
GR: Griess reagent
TPA: 12-O-tetradecanoylphorbol-13-acetate
CT26: The murine colorectal cancer cell line
J774A.1: The murine macrophages cell line
IL-1, IL-6, IL-10: Interleukin 1, 6 and 10
TNF-α: Tumor necrosis factor- α
CDDP: Cisplatin
IC_50_: Half maximal inhibitory concentration
NO: Nitric oxide
ED_50_: Median effective dose
